# Successful Management of a Giant Mucinous Cystadenocarcinoma of the Ovary Through Laparotomy: A Case Report

**DOI:** 10.7759/cureus.60474

**Published:** 2024-05-16

**Authors:** Rajasi K Sengupta, Charu Pareek, Ankit Badge, Pravin W Nikhade

**Affiliations:** 1 Gynecology, Datta Meghe Medical College, Datta Meghe Institute of Higher Education and Research (Deemed to Be University), Nagpur, IND; 2 Clinical Embryology, Datta Meghe Medical College, Datta Meghe Institute of Higher Education and Research (Deemed to Be University), Nagpur, IND; 3 Microbiology, Datta Meghe Medical College, Datta Meghe Institute of Higher Education and Research (Deemed to Be University), Nagpur, IND; 4 Surgery, Datta Meghe Medical College, Datta Meghe Institute of Higher Education and Research (Deemed to Be University), Nagpur, IND

**Keywords:** bilateral salpingo-oophorectomy, total abdominal hysterectomy, laparotomy, giant cystadenocarcinomas, ovarian cancer

## Abstract

Giant mucinous cystadenocarcinoma of the ovary is rarely described. Huge ovarian masses are mostly benign, but malignancy should be ruled out by investigations and clinical assessment. Here, we present a case of a large mucinous cystadenocarcinoma of the ovary in a 48-year-old postmenopausal woman. Imaging examinations revealed a large cystic tumor that filled the whole abdominal cavity. Despite the difficulties presented by the size of the tumor and its malignant potential, laparotomy was carried out, which included bilateral salpingo-oophorectomy, total abdominal hysterectomy, exploration of other intra-abdominal organs, and pelvic lymphadenectomy. Histopathology indicated the presence of mucinous cystadenocarcinomas. Adjuvant chemotherapy was given post-operatively, and the patient maintained remission during follow-up. This case emphasizes the need for early detection by simple imaging modalities such as ultrasonography in cases of ovarian masses. Most adnexal masses, if detected early, are amenable to surgical management with a good prognosis. Large masses underline the need for a multidisciplinary approach to improve patient outcomes.

## Introduction

Giant ovarian tumors, defined as ovarian masses that are considered large and have diameters between 5 cm and 15 cm, or when tumors exceed 20 cm in size, have become increasingly rare due to advances in early detection and treatment. Among these tumors, serous types are more prevalent than mucinous ovarian carcinoma [1]. It accounts for 10% to 15% of all benign ovarian tumors [2]. Mucinous ovarian carcinoma was believed to constitute approximately 12% of ovarian malignancies. However, recent estimations show the true incidence to be approximately 3%. The majority of patients with these tumors are females in their 20s and 40s. However, cases have also been reported in adolescents, premenarchal girls, and postmenopausal patients [3]. Although mucinous cystadenocarcinomas can develop in any organ, they are most frequently linked to the appendix and the ovaries. Though less frequently, they can also be discovered in other organs such as the stomach, colon, and pancreas.

Benign mucinous cystadenoma, mucinous tumors exhibiting borderline, pseudomyxoma peritonei, or minimally malignant potential, and invasive mucinous ovarian cancer are examples of ovarian mucinous tumors [4]. These huge-sized tumors often exert pressure on neighboring organ systems, leading to compression symptoms, particularly affecting the urinary and respiratory systems. For more than 40 years, the primary diagnostic tool for ovarian cancer has been the tumor biomarker cancer antigen 125 (CA125). The emphasis on diagnostic biomarkers using CA125 to identify ovarian cancer in stages I and II has not increased patient survival. Type II tumors are responsible for the great majority of ovarian cancer mortality, based on the dual model of ovarian cancer carcinogenesis. Yet, stage I and II type II cancers are uncommon [5]. The main objective of this study is to draw attention to big ovarian epithelial cysts that present unexpectedly to minimize the possibility of misdiagnosis, incorrect diagnosis, and poor treatment.

## Case presentation

We present the case of a 48-year-old postmenopausal female with a history of abdominal fullness and escalating dyspnea over the past three months. She was para 4, live 4 (P4L4), all full-term normal delivery (FTND), and had attained menopause five years ago. She had no history of any malignancy. The patient was obese, with a pulse rate of 76 beats per minute and a blood pressure of 130/80 mmHg. Per the abdomen examination, it was observed to be tense and distended. Per speculum, the cervix appeared hypertrophied and congested, although the vagina appeared healthy. However, during a per-vaginal examination, the uterus could not be palpated due to a cystic mass that filled the whole abdomen and pelvis.

Table 1 shows the results of the preoperative laboratory tests: hemoglobin (Hb) level was 10.2 g/dL, bleeding time (BT) was 5 minutes, clotting time was 10 minutes, platelets were 425,000/mm^3^, the erythrocyte sedimentation rate (ESR) was 43 mm at the end of the first hour, and serum CA125 was 66.57 U/mL.

**Table 1:** Laboratory test results g: gram; dL: deciliter; mm: millimeter; hr: hour; U: unit; mL: milliliter; ESR: erythrocyte sedimentation rate; CA: cancer antigen

Laboratory Findings	Results	Reference Values
Hemoglobin	10.2 g/dL	12.0-15.5 g/dL
Platelets	425,000/mm³	150,000-400,000/mm³
ESR	45 mm/hr	0-20 mm/hr
Serum CA125	66.57 U/mL	<35 U/mL

Table 2 shows the ascitic fluid biochemistry as follows: total leukocyte count (TLC) 35/mm^3^, lymphocytes 70%, cobweb absent, proteins 3.9 mg/dL, and ascitic fluid cytology: negative for malignancy.

**Table 2:** Ascitic fluid biochemistry mm: millimeter; mg: milligram; dL: deciliter

Ascitic Fluid Biochemistry	Results
Total leukocyte count	35/mm^3^
Lymphocytes	70%
Cobweb appearance	Absent
Total proteins	3.9 mg/dL
Ascitic fluid cytology	Negative for malignancy

On examination

**Figure 1:**
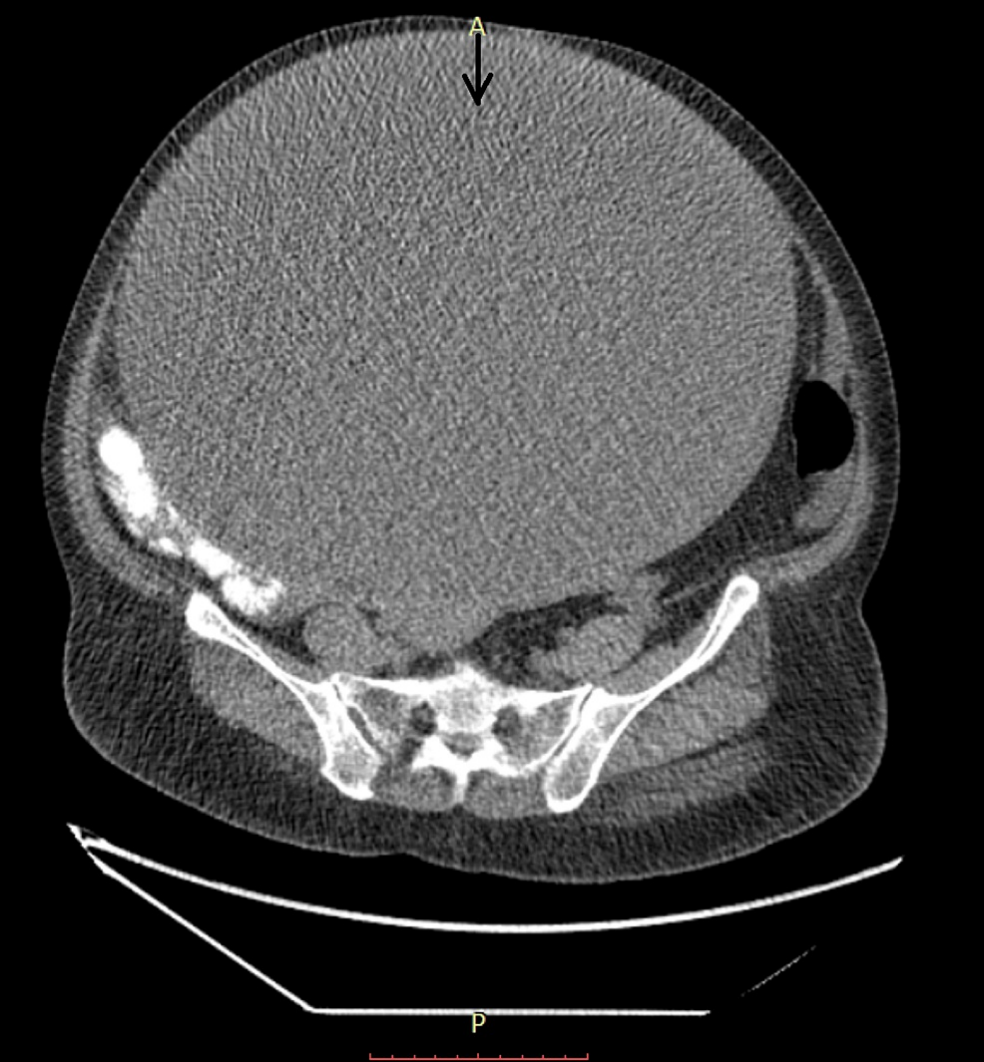
Abdominal CT scan showing a giant ovarian cyst The black arrow shows the ovarian cyst. A: anterior side; P: posterior side; CT: computed tomography.

A huge cystic mass occupied the whole abdomen, and the uterus was not palpable on pelvic examination. A computed tomography (CT) scan was advised to diagnose the condition, and its impressions showed a large cystic lesion arising from the pelvis extending into the upper abdomen. The dimensions of the lesion are size 36 x 34 x 25 cm, with a wall thickness of 6.5 mm, a dependent density pattern, and a few enhancing septae. There is a small, solid, calcified component measuring 3.3 x 2.3 cm on the right posterior of the cyst. The cyst is causing the peripheral displacement of bowel loops. The anterior indentation is causing a thinning of the rectus muscles over the anterior abdominal muscles. Fat planes between cysts and surrounding structures are well-maintained, as shown in Figure 1.

**Figure 2:**
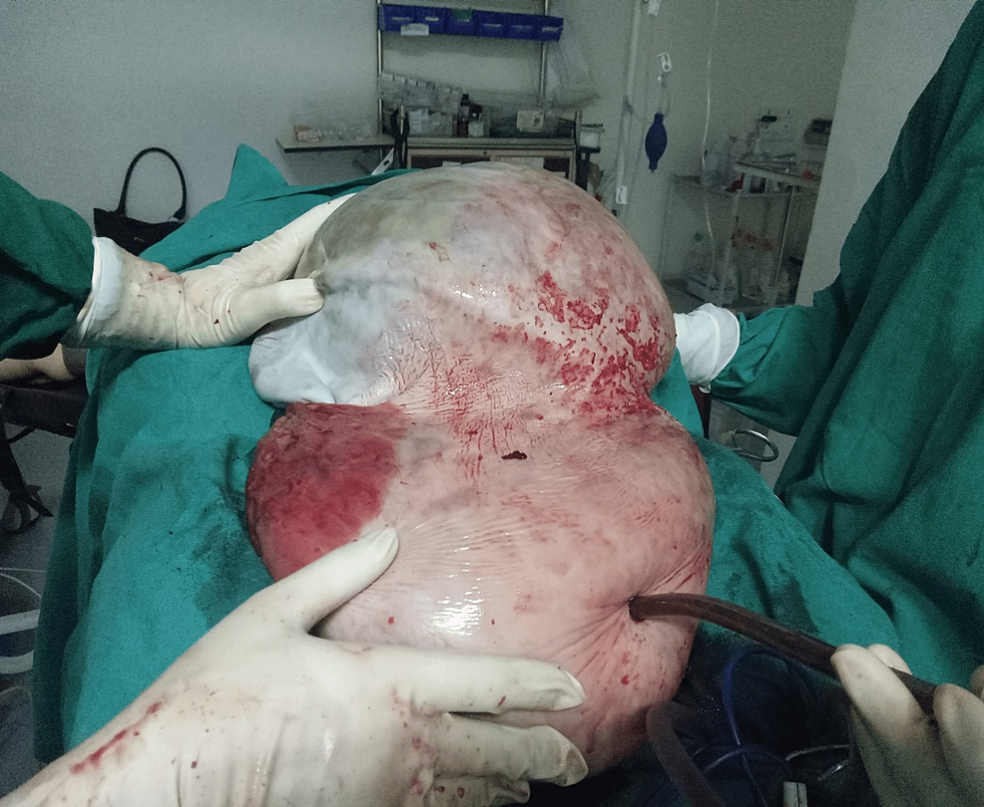
Intraoperative incision during laparotomy

A laparotomy was conducted under general anesthesia. It revealed a voluminous cyst tumor filling up the entire abdominal cavity. A midline incision was made up to 10 cm above the umbilicus, as shown in Figure 2. Following the incision, a large left ovarian tumor was discovered. The uterus, omentum, right ovary, and peritoneum seemed normal, with no malignant seedings. Total abdominal hysterectomy with bilateral salpingo-oophorectomy, pelvic lymphadenectomy, partial omentectomy, and exploration of other intra-abdominal organs such as the liver and kidneys was successfully performed.

**Figure 3:**
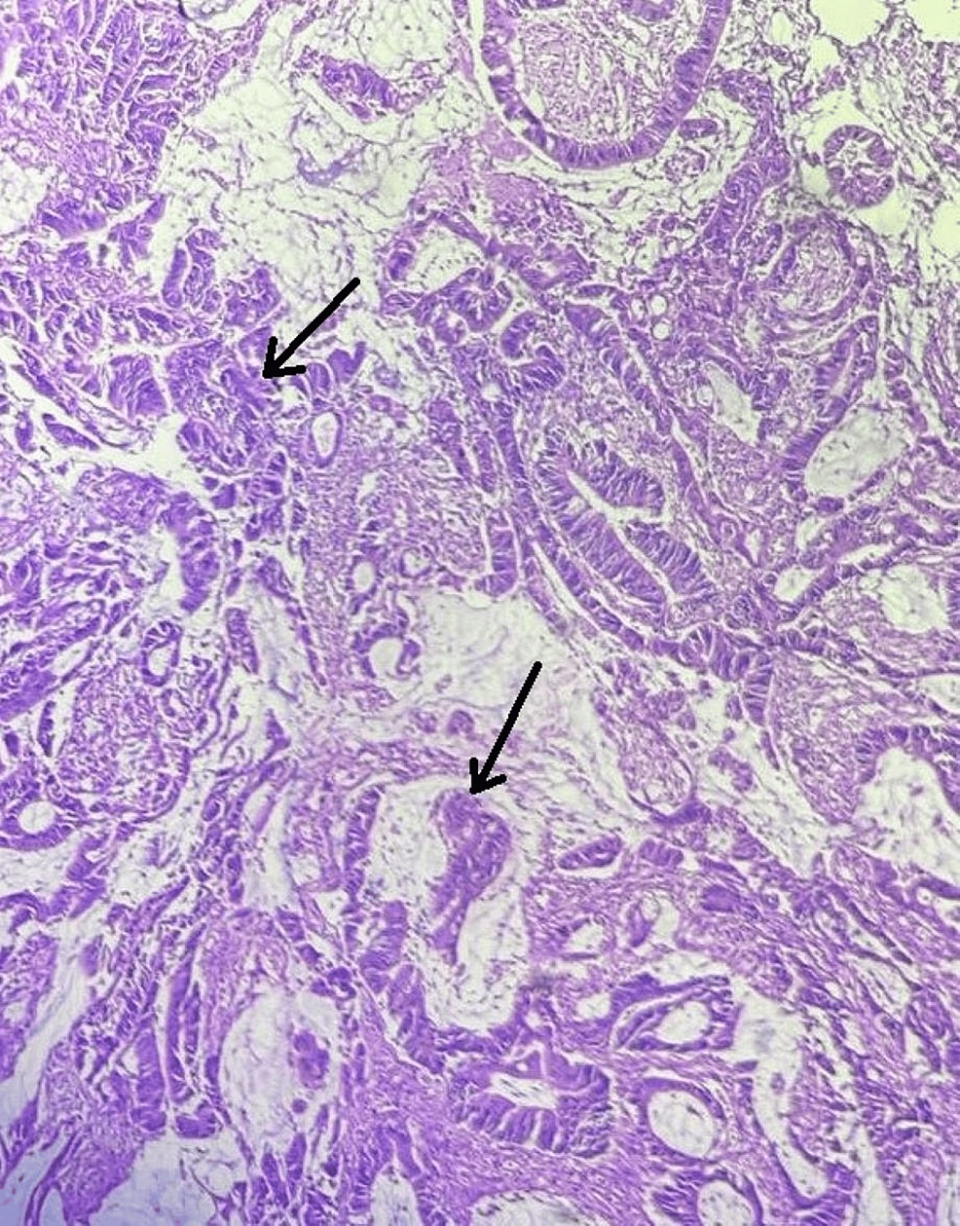
Histopathological analysis of mucinous cystadenocarcinoma The black arrows show the mucinous cystadenocarcinoma of the ovary.

The patient's hemodynamic status was stable until the completion of the surgery. The operational time was two hours and 15 minutes, and approximately 26 L of altered blood-mixed fluid was drained. All the specimens were taken for pathological investigation after the surgical removal of the cyst. Histopathology analysis confirmed the diagnosis of mucinous cystadenocarcinoma, grade II, weighing 30.1 kg with a size of 36 x 34 x 25 cm, arising from the left ovary with the left fallopian tube stretched over it (Figure 3). Despite the negative ascitic fluid cytology, the patient received adjuvant treatment after six cycles of chemotherapy treatment and remained in remission during follow-up.

## Discussion

A study conducted by Katke [6] showed that an older woman had an undetected malignant large abdominal tumor. Although the cystadenocarcinoma had a unique look and an unexpected diagnosis, it was successfully removed without spilling or spreading. Rare reports of giant ovarian cystadenocarcinomas have been noted. Large ovarian tumors tend to be benign, but it is important to rule out cancer through testing and clinical assessment. Due to their compressing symptoms or the possibility of cancer, giant cysts must be removed. A laparotomy is always required as part of the treatment to prevent perforation and cyst fluid leakage to the peritoneal cavity. The 48-year-old female patient, who underwent exploratory laparotomy to remove a cystic tumor, experienced severe and rapidly growing abdominal distension, as described by the author. The tumor was diagnosed as metastatic mucinous cystadenocarcinoma with omental metastases after histological examination. Issues with diagnosis and treatment are discussed in light of the surprising and unusual appearance of this ovarian cystadenocarcinoma [6].

In their study, Grigore et al. [7] concluded that there is a relatively limited number of instances and the dispersed availability of information that limits the scope of analysis based on statistics. Although rare, huge ovarian cysts can occur during adolescence and are usually benign. To get a better surgical outcome, it is better to first evaluate the results of imaging and tumor markers. Then, the use of laparoscopic surgery should be evaluated as a viable alternative, particularly if there are no predicted factors for malignant disease [7].

Brown and Frumovitz [8] stated that, based on a clinical, histologic, and molecular perspective, these tumors are closely related to one another yet different from all other histological subtypes of epithelial ovarian neoplasms. Mucinous tumors of the ovary include benign mucinous cystadenoma, pseudomyxoma peritonei, mucinous tumors with minimal malignant potential (borderline), and invasive mucinous ovarian cancer. Mucinous ovarian tumors are a separate histologic entity. They differ greatly from other forms of epithelial ovarian tumors in terms of pathophysiology, pathological traits, and clinical presentation. These disparities highlight the need for novel therapeutic techniques to enhance patient outcomes [8].

Uccella et al. [9] noted that the application of pre-operative laparoscopic investigation and intra-operative ultrasound may boost the capacity to choose patients while also improving the ability to drastically resect the disease without opening the abdomen using a wide surgical incision. Cancer of the ovaries remains a severe diagnostic and therapeutic issue. Minimal-invasive surgery should be considered as a viable option over laparotomy for surgical secondary cytoreduction of recurrent ovarian cancer in carefully chosen cases, at specialized oncological centers, and maybe in the framework of well-conducted scientific studies [9].

The patient in our study required immediate medical attention due to the sudden presentation of severe stomach pain and distention. A palpable mass and an unusual increase in abdomen circumference raised concerns about an ovarian malignancy. In this instance, imaging tests, particularly contrast-enhanced CT, were essential in locating the ovarian tumor. A complex cystic tumor in the left ovary and cystadenomas with internal septations, solid components, and calcification were found by the CT scan. The surgical plan and choice for a bilateral salpingo-oophorectomy and subsequent total abdominal hysterectomy were based on this information.

## Conclusions

Giant mucinous cystadenocarcinoma of the ovary presents unique diagnostic and intervention challenges. This case highlights the significance of recognizing and promptly investigating abdominal symptoms, even in cases with a relatively brief history. The extremely large size of the tumor underscores the importance of early detection and intervention. If diagnosed at an earlier stage, the treatment is complete, quick, and easy, resulting in a favorable prognosis for patients and an improved five-year survival rate. A massive cystic tumor filling the entire abdominal cavity necessitates surgical intervention. This radical surgical management addresses the extent of the tumor and mitigates its potential risks. The limitation of relying solely on CA125 values and ascitic fluid cytology is emphasized, as they may not always accurately reflect the presence of malignancy. Multidisciplinary care is recognized as the best practice in treatment planning and care for patients.
